# Recombinant protein embedded liposome on gold nanoparticle based on LSPR method to detect Corona virus

**DOI:** 10.1186/s40580-023-00399-x

**Published:** 2023-10-30

**Authors:** Lina Kim, Seongjae Jo, Gyeong-Ji Kim, Kyung Ho Kim, Sung Eun Seo, Eunsu Ryu, Chan Jae Shin, Yu Kyung Kim, Jeong-Woo Choi, Oh Seok Kwon

**Affiliations:** 1 Infectious Disease Research Center, Korea Research Institute of Bioscience and Biotechnology (KRIBB), Daejeon, 34141 Republic of Korea; 2 Department of Chemical & Biomolecular Engineering, Sogang University, Seoul, 04107 Republic of Korea; 3 Department of Clinical Pathology, School of Medicine, Kyungpook National University, Daegu, 41944 Republic of Korea; 4 SKKU Advanced Institute of Nanotechnology (SAINT), Sungkyunkwan University (SKKU), Suwon, 16419 Republic of Korea; 5 Department of Nano Science and Technology, Sungkyunkwan University (SKKU), Suwon, 16419 Republic of Korea; 6 Department of Nano Engineering, Sungkyunkwan University (SKKU), Suwon, 16419 Republic of Korea

**Keywords:** Virus sensor, Protein embedded liposome, Recombinant protein, SARS-CoV-2 detection, LSPR

## Abstract

Antibody sensor to detect viruses has been widely used but has problems such as the difficulty of right direction control of the receptor site on solid substrate, and long time and high cost for design and production of antibodies to new emerging viruses. The virus detection sensor with a recombinant protein embedded liposome (R/Li) was newly developed to solve the above problems, in which R/Li was assembled on AuNPs (Au@R/Li) to increase the sensitivity using localized surface plasmon resonance (LSPR) method. Recombinant angiotensin-converting enzyme-2 (ACE2) was used as host receptors of SARS-CoV and SARS-CoV-2, and the direction of enzyme active site for virus attachment could be controlled by the integration with liposome. The recombinant protein embedded liposomes were assembled on AuNPs, and LSPR method was used for detection. With the sensor platform S1 protein of both viruses was detected with detection limit of 10 pg/ml and SARS-CoV-2 in clinical samples was detected with 10 ~ 35 Ct values. In the selectivity test, MERS-CoV did not show a signal due to no binding with Au@R/Li. The proposed sensor platform can be used as promising detection method with high sensitivity and selectivity for the early and simple diagnosis of new emerging viruses.

**Graphical Abstract:**

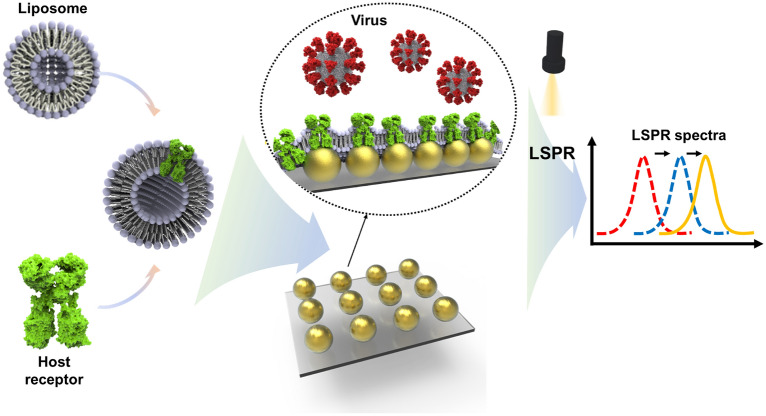

**Supplementary Information:**

The online version contains supplementary material available at 10.1186/s40580-023-00399-x.

## Introduction

The World Health Organization declared coronavirus disease (COVID-19) a pandemic in December 2019, following the initial outbreak of severe acute respiratory syndrome coronavirus-2 (SARS-CoV-2). Worldwide, SARS-CoV-2 infection has been reported in nearly 626 million people, and over 6.6 million deaths have been reported as of November 2022 [1]. SARS-CoV-2 causes respiratory disease through a disease-inducing process similar to that of SARS-CoV-1, which was prevalent in 2002, and Middle East respiratory syndrome (MERS)-CoV, which was prevalent in 2012 [2]. The World Health Organization announced in February 2018 that unknown future infectious diseases will occur that we do not already know. The recent spread of COVID-19 virus became the first case of disease X [3]. Despite the complexity of pathogens, limitations of human knowledge, and uncertainty of scientific facts, countermeasures are needed. Diagnosis techniques are prepared to prevent high risk of any viral infection. Generally, diagnostic tools for detecting virus infection use antigen–antibody tests and real-time polymerase chain reaction (RT‒PCR) [4]. Rapid antigen tests using the lateral flow assay (LFA), which is the most accessible diagnostic test, provide diagnosis results within 15 min after loading nasal or saliva samples [5]. However, the result of LFA caused confusion due to high false positive/false negative rates, and the accuracy of LFA in detecting a small amount of virus is lower than that of PCR. RT‒PCR gives the most accurate infection diagnosis results by amplifying and quantifying the nucleic acid of a sample suspected of virus infection [6]. The limitations of PCR technology include the requirement of a complex extraction step for viral ribonucleic acid (RNA) and a long detection time [7]. Therefore, there is an urgent need for the development of an advanced detection platform that is fast and has a high sensitivity and low false-negative rate to eradicate the spread of the virus.

Several studies have been reported on identifying unknown pathogens using recognition elements, such as receptors [8], characteristics such as individual features or antibiotic inhibition patterns [9] and nucleic acid probes [10]. For virus detection, the monoclonal antibodies (mAbs) and multi-specific antibodies are used for elements [11]. However, expression of antibodies is ineffective than recombinant protein in *Escherichia coli* (*E. coli*). Angiotensin-converting enzyme 2 (ACE2) has been studied as a receptor candidate for transmembrane cellular receptors to bind to SARS-CoV and SARS-CoV2, and these receptors mediate viral entry into cells [12]. Additionally, many studies have been reported on using the ACE2 receptor for the treatment or diagnosis of COVID-19 [13]. However, receptors alone are structurally unstable on the substrate and can be easily unfolded under the action of the external environment [14]. Therefore, our study suggests developing liposomes embedded in cell-derived ACE2 receptors as platform that improve the stability and functionality of the ACE2 receptors to detect low concentrations of COVID-19 in real time. Our platform can be applied to detect unknown pathogens without gene sequencing.

Liposomes compose the bilayer membrane and are spontaneously formed by the dispersion of phospholipids in an aqueous solution [15]. Additionally, the properties of liposomes include low toxicity, biocompatibility, the ability to entrap both hydrophobic and hydrophilic materials, and an abundance of negatively and positively charged molecules [16]. Thus, liposomes play important roles as ideal carriers of antigens, drugs, and active ingredients [17]. Moreover, several studies reported that liposome supported gold nanoparticles with properties such as nano delivery and used as multifunctionalized biomedical sensor [18]. In this study, AuNP was used as a substrate tool to enhance the sensitivity of a localized surface plasmon resonance (LSPR) sensor to detect the binding between COVID-19 and the ACE2 receptor.

This study newly developed liposomes embedded with recombinant ACE2 receptor to enhance the sensitivity of COVID-19 detection and applied them in optical sensors such as LSPR sensors for real-time monitoring and rapid detection without preprocessing. Liposome-functionalized LSPR sensor never been done to detect SARS-CoV and SARS-CoV-2. Considering all properties of liposomes embedded with recombinant receptor, we believe that this platform has potential as a simple and innovative material for detecting various pathogens and viruses in ultra-trace amounts in early stages in clinical samples without complex processing.

## Methods/experimental

### Materials

Bacto™ Yeast Extract, Bacto™ Tryptone from Gibco, *E. coli* BL21(DE3) cell, PBS (phosphate buffered saline) pH (7.4), PVDF, which is transfer membrane, of a 0.45 μm size were obtained from Thermo Fisher Scientific, Waltham, MA. Sodium chloride was obtained from Junsei, Japan for LB (Luria–Bertani) media. Ampicillin sodium salt, Triton X-100, 2-mercaptoethanol, TEMED (tetramethylethylenediamine) were obtained from Sigma‒Aldrich (St. Louis, MO, USA). IPTG (isopropyl β-D-thiogalactopyranoside) from LPS solution were purchased for protein expression. Ultrasonic liquid processors for cell lysis (Sonics vibra VCX 500), an ultracentrifuge (Hanil, ultra5.0), a shaking incubator (JSR, JSSI-100 T), FPLC for affinity chromatography (Cytiva, AKTA pure™), an Amicon® Ultra- centrifugal filter with 10 kDa from Millipore, and a clean bench for bacteria expression (JSR) were purchased for protein purification. SDS‒PAGE (sodium dodecyl polyacrylamide gel electrophoresis) and western blotting were conducted to detect protein bands by molecular weight with a Mini Trans-Blot cell from Bio-Rad, 10X TBS-T from LPS solution, 30% acrylamide/bis solution 29:1 from Bio-Rad, 6X His tag monoclonal antibody for the 1st antibody, and goat-anti mouse IgG horseradish peroxidase for the 2nd antibody from Invitrogen. For liposome synthesis, POPC and POPG from Avanti Polar Lipids (Alabaster, AL, USA) were mixed and extruded by a mini-extrusion instrument from Avanti Polar Lipids. SARS-CoV-2 S1 protein (40591-V08H; Sino Biological, Inc., China), MERS-CoV S1 protein (40069-V08H), and SARS-CoV-2 S1 protein (alpha, beta, kappa variants, 40591- V08H-12, 10, 21) were purchased for standard sample experiments.

### Expression and purification of ACE2

The pET21a ( +) vector was used for ACE2 cloning, and the *E. coli* BL21(DE3) cells were expressed. The cells were cultured in LB medium with ampicillin and incubated with 10 mM IPTG overnight at 16 °C until the OD600 (optical density (600 nm)) reached from 0.5 to 1.0. For the purification of ACE2, cells were incubated in PBS (pH 7.4) and 0.1 M PMSF (phenyl methane sulfonyl fluoride) and then incubated for 5 min at 4 °C. The suspension was sonicated on and off in 5 s pulses for 30 min with an ultrasonic liquid processor and ultracentrifuged at 12,000*g* for 20 min. The supernatant was filtered with a 0.45 µM filter. Then, the supernatant was inserted into a Ni–NTA column with 5 mL of HisTrap HP and eluted with imidazole buffer using an affinity chromatography mechanism. Three different buffers were used: 50 mM Tris–HCl (pH 8.0) and 0.5 M NaCl, 50 mM Tris–HCl (pH 8.0) and 0.5 M NaCl 20 mM imidazole, and 50 mM Tris–HCl (pH 8.0) and 0.5 M NaCl 250 mM imidazole.

### Characterization of ACE2

After purification, the buffer containing imidazole was changed by dialysis at 4 °C overnight. SDS‒PAGE and western blotting were conducted on the obtained samples for analysis using 10% polyacrylamide amide gels. Coomassie blue staining buffer was used to confirm the band without an antibody. Before western blotting, PVDF membrane, the protein was transferred, was blocked with BSA (bovine serum albumin) for 30 min at 37 °C and 100 rpm in a shaking incubator and incubated with antibody. Chemiluminescence detection was carried out for detection the protein band. When the band was detected at 85 kDa molecular weight and available to select specific protein, an amicon tube (0.5 mL, 10 kDa cutoff) was collected to obtain a high concentration of protein. Then, the protein was calculated with an albumin standard for BCA (Bicinchoninic acid) Pierce in a protein concentration assay.

### Liposome preparation

The phospholipids were dissolved in chloroform (1 mg/mL), and after dissolving the lipids, samples were evaporated by argon gas for 4 h to 18 h. The lipid film was constructed after evaporation and dissolved in HEPES buffer (pH 8.0), and the solution buffer was changed by dialysis for 5 h by Slide-A-Lyzer™ G2 Dialysis Cassettes (3.5 K MWCO, 30 mL). Liposomes were prepared using the sonication method and mini extruder. Sonication was performed for 30 min, and a mini extruder with 15 passes made liposomes 100–200 nm with a 100 nm pore size in a polycarbonate membrane.

### Reconstitution of recombinant protein with liposomes

The purified ACE2 was mixed with a ratio of R:POPG of 1:6000. To remove detergent, biobeads were used for reconstitution. Subsequently, sonication in ice and incubated for 1 h was performed to resize the liposomes. R/Li was inserted onto a Superdex 200 Increase 10/300 GL size exclusion column, and the size was determined by DLS.

### Synthesis of YFP with ACE2

The YFP gene was cloned into the N-terminal ACE2 gene with a 6-histidine tag. Before transforming the gene in bacteria, the gene was amplified for polymerase chain reaction. After amplification, the size of the gene was checked by DNA electrophoresis with pfu DNA polymerase and a 1% agarose gel. The gel with bands was purified with a gel purification kit. Restriction enzyme treatment was performed for 5 h, and the ligation step was performed for 2 h. The result was checked with DNA electrophoresis again with 2X PCR master mix. The colony was transformed into *E. coli* (DH5a) for colony PCR. The colony was extracted with a mini-prep kit, and then the commission of the sequencing was requested.

### Biosimulation of POPC, POPG with ACE2 and antibodies

For docking analysis, the structure of POPC was drawn by ChemDraw and Chem 3D. The structures of the ACE2-spike protein of the SARS-CoV-2 complex (PDB ID: 6M0J), SARS-CoV (PDB ID: 2AJF), CR3022 antibody (PDB ID: 6XC3), PB2-2F6 (PDB ID: 7BWJ), REGN10933 (PDB ID: 6XDG) were obtained from the Protein Data Bank. The POPC,POPG complex and ACE2-spike protein complex were docked using AutoDock 4.2 and CHARMM-GUI. By changing the coupling structure, Autodock software calculated the energy and explored the structure in which the energy was minimized. Using an energy model, it also predicted the binding free energy (in other words, the binding force, affinity, and binding scaling) by Pymol (The PyMOL Molecular Graphics System, Version 1.2r3pre, Schrödinger, LLC).

### Synthesis of AuNPs for LSPR sensors

For regular size and shape of AuNPs, chemical redox reaction with HAuCl_4_ solution were added with citric acid for seed solution. After the process, same step was repeated for growth step. To calculate size and shape of final products of AuNPs, TEM and DLS analysis were conducted.

### Clinical sample preparation

The clinical samples for the LSPR sensor (samples of each representative (cycle threshold) Ct value range: from 10 to 35) were obtained from Kyungpook National University Hospital (IRB approval number DGIRB 2021-05-003-001). To obtain samples for detection, saliva was collected in universal transport medium (UTM).

## Results and discussion

### Schematic illustration of Au@R/Li

**Fig. 1:**
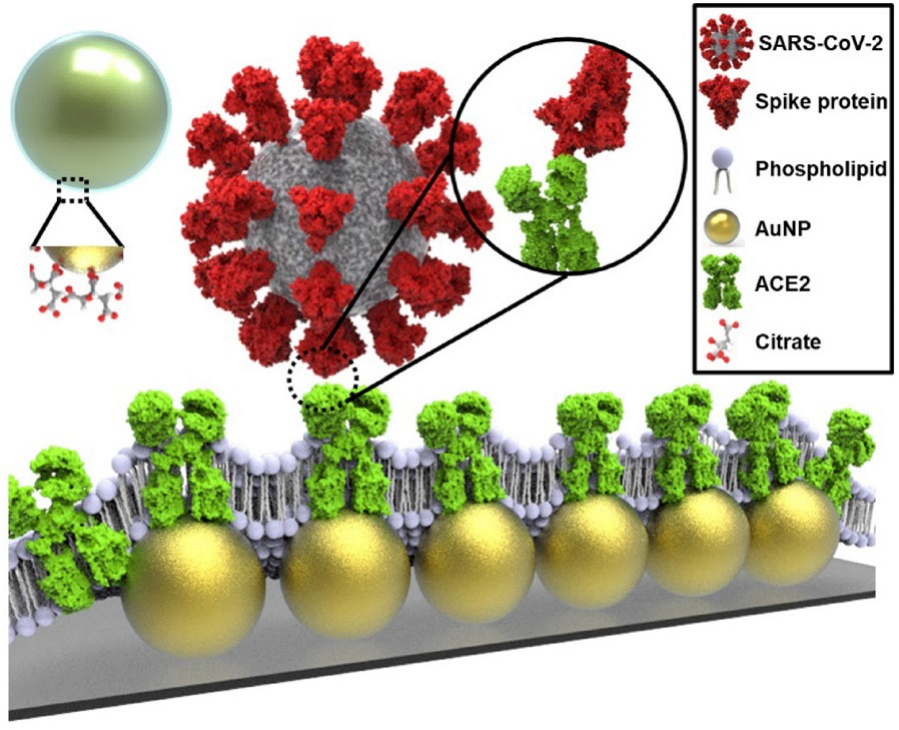
Schematic illustration of virus detection by recombinant protein embedded liposome (R/Li) on AuNPs (Au@R/Li)

The AuNPs, which were surrounded by a citrate group, formed patterned arrays on glass. For biosensors, nanomaterials, including AuNPs, are specialized for enhanced detection with high sensitivity and selectivity [19]. Using nanomaterials has great advantages in applications targeting DNA, RNA, proteins, and other small ligands/molecules [20]. LSPR biosensors with AuNPs have the advantage of being able to detect small analytes and have been successfully applied in clinical diagnostics [21]. A shift in the absorption peak when the receptor bound to the target was measured with UV‒Vis spectra [22]. Detection of the SARS-CoV-2 spike protein was achieved by applying an LSPR sensor mechanism. The spike protein of SARS-CoV-2 binds to the ACE2 receptor to infect and penetrate in cell entry [23]. As shown in Fig. 1, to detect the spike protein, the binding receptor embedded in liposomes covered the AuNPs. The inset depiction of phospholipids on AuNPs is presented in the supplementary information. (Additional file 1: Fig. S1) The ACE2 receptor, which is a transmembrane protein, is usually embedded in the lipid bilayer via strong hydrophobic interactions with phospholipids [24]. ACE2 receptors reconstituted in liposomes (e.g., R/Li) performed similarly to the receptors in natural membrane systems. In addition, R/Li is useful for antibody detection, functional studies, and drug measurement [25]. AuNPs surrounded with liposomes (e.g., Au@R/Li) immobilize the ACE2 protein on the surface; thus, sensitivity and stability are improved.

To achieve this objective, we conducted the characterization of R/Li, optimization of Au@R/Li, and sensing experiments of standard samples and clinical samples.

### Characterization of the ACE2 receptor and liposomes

**Fig. 2:**
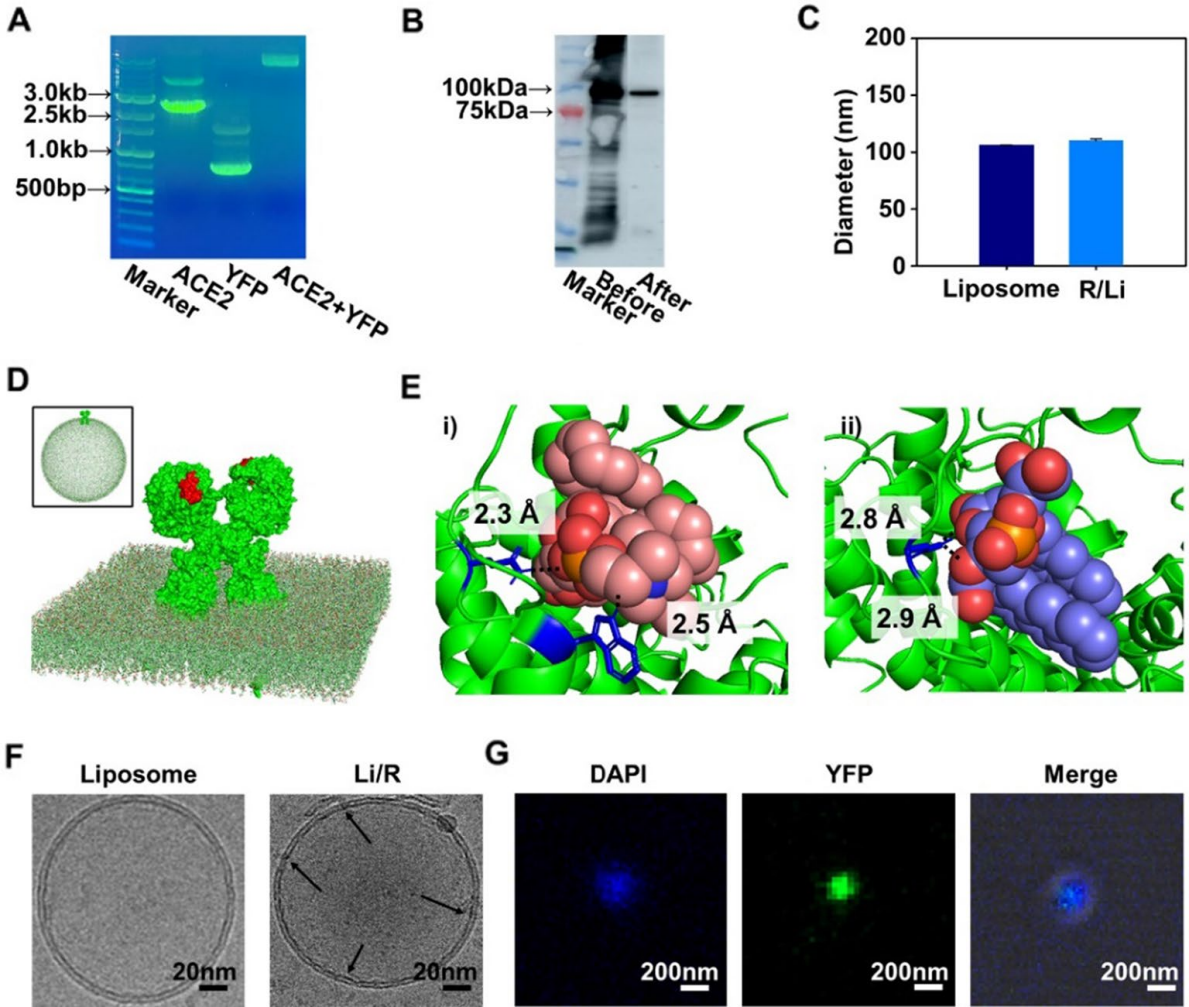
Preparation and Characterization of ACE2 and R/Li. **A** Confirmation of vector and insert by ACE with YFP. **B** Western blot for recombinant protein expression image of ACE2. (Before/After purification) (**C**) Size distribution of DLS depending on sonication time and extrusion of liposomes and R/Li. **D** Result of representation ACE2 in membrane by ChARMM-GUI. (Inlet = Actual representation of ACE2 in liposome) The binding domain with SARS-CoV-2 S1 protein was shown as red. **E** Simulation of ACE2 with POPC and POPG by AutoDock. **F** Cryo-EM images of liposomes (left) and R/Li (right). (Scale bars = 20 nm) **G** Confocal microscope image of R/Li with YFP (yellow fluorescence protein). Scale bars = 200 nm (Arrow = receptor)

The recombinant protein ACE2 was encoded in pET21a ( +) with a 6His tag in the N-terminus of the sequence. After cloning, host cell is *E. coli* (BL21-DE3) for the mass production and high-quality expression of proteins under simple culture conditions. To use ACE2 for receptor diagnosis, *E. coli* was expressed for gene cloning. Therefore, *E. coli* was used for protein expression because *E. coli* has the ability to grow faster than other hosts and can easily express proteins [26]. Due to these characteristics, ACE2, which is expressed in *E. coli*, was synthesized for SARS-CoV-2 detection. The yellow fluorescence protein (YFP) sequence was attached to the sequence of ACE2 preceding the 6His amino acid. Figure 2A shows the combination of the ACE2 protein and histidine amino acid tag through gel electrophoresis, UV‒Vis, and western blot analysis. The size of the ACE2 gene is approximately 2.4 kb, and that of YFP is 1 kb. It is essential to confirm the insertion by plasmid size for the connection of ACE2 and YFP with agarose electrophoresis. After confirmation of insertion of YFP in ACE2 in the vector, a clear western blotting image of ACE2 at 85 kDa was obtained, as shown in Fig. 2B. The expression of ACE2 was confirmed, and the difference was compared before purification (left) and after ACE2 purification (right). In the case of proteins with histidine in the terminal sequence, a specific protein was selected using affinity chromatography, which used the mechanism of correlation between histidine and nickel [27]. Accordingly, it was shown that the properties of the recombinant protein and specific protein that was needed were selectively obtained. To synthesize liposomes, purified ACE2 was assembled and embedded into liposomes by optima ratio (Additional file 1: Fig. S2). Size distribution analysis was performed with dynamic light scattering (DLS), which was helpful for calculating size of liposomes in Fig. 2C. To manipulate size of liposomes, an extrusion filter and sonication (Additional file 1: Fig. S3). For comparison of the bare liposome and R/Li, the receptor was reacted with the bare liposome, and there were a few changes in the shape or size of the liposome, which indicated the stability of the liposome and confirmed that the protein and liposome were synthesized well. Following the synthesis step, the diameter of the receptor-embedded liposome increased to 106 ± 1.24 nm from the 102 ± 0.45 nm diameter of the bare liposome. After measuring the random changes in the intensity of light scattered to measure the particle size, a simulation by CHARMM-GUI [28, 29] and AutoDock [30] software confirmed the mechanism in which the receptor was embedded in the liposome in Fig. 2D, E. (i) The distance was calculated, and the binding affinity of ACE2 and 1-palmitoyl-2-oleoyl-sn-glycero-3-phosphocholine (POPC) allowed the interacting ligand and protein to be docked together. The binding affinity was calculated to be − 6.8 kcal/mol. The binding distance between the residue GLN 145 of ACE2 and the POPC complex was 2.3 Å, and that of TRP 742 was 2.5 Å. In addition, the interactions of GLN 145 and TRP 742 with POPC included hydrogen bonding, and hydrophobic interactions occurred with other residues (Additional file 1: Fig. S4). (ii) The distance was calculated, and the binding affinity of ACE2 and 1-palmitoyl-2-oleoyl-sn-glycero-3-(phospho-rac-(1-glycerol) (POPG) was calculated. The binding affinity was calculated to be − 6.3 kcal/mol. There were hydrogen interactions with GLN 736 at 2.8 Å and 2.9 Å. Interactions were confirmed and predicted through possible binding positions and binding structures based on structural information of phospholipids, which are components of liposome and ACE2 by simulation. To determine the difference between protein and antibody, simulation of binding affinity of antibodies was carried out (Additional file 1: Fig. S5). The binding affinity of CR3022 antibody with liposome was − 3.8 kcal/mol with POPC, − 5.3 kcal/mol with POPG. In REGN10933 antibody, binding affinity was − 4.4 kcal/mol with POPC, − 5.8 kcal/mol with POPG. For PB2-2F6, there was − 3.7 kcal/mol in POPC and − 4.3 kcal/mol in POPG. ACE2 has higher binding affinity than antibodies in liposome. Moreover, structures were predicted when liposomes and proteins interact by CHARMM-GUI. For ACE2, it was confirmed that liposome did not interfere binding site while had difficulty to control direct direction of binding site in antibodies. The reconstitution of membrane proteins into liposomes was validated with cryogenic electron microscopy (Cryo-EM) in Fig. 2F. According to the results, comparing the sizes of bare liposomes (left) and R/Li (right) showed that the protein was located between the membrane spaces. Figure 2G shows a confocal image verifying the insertion of YFP tagged on the ACE2 protein into liposomes. Bare liposomes were dyed with DAPI (ex359/em457), and R/Li was synthesized with YFP (ex513/em530). The merged image demonstrated that the developed ACE2 protein was well purified and embedded correctly on the liposome surface.

### Characterization of Au@R/Li

**Fig. 3:**
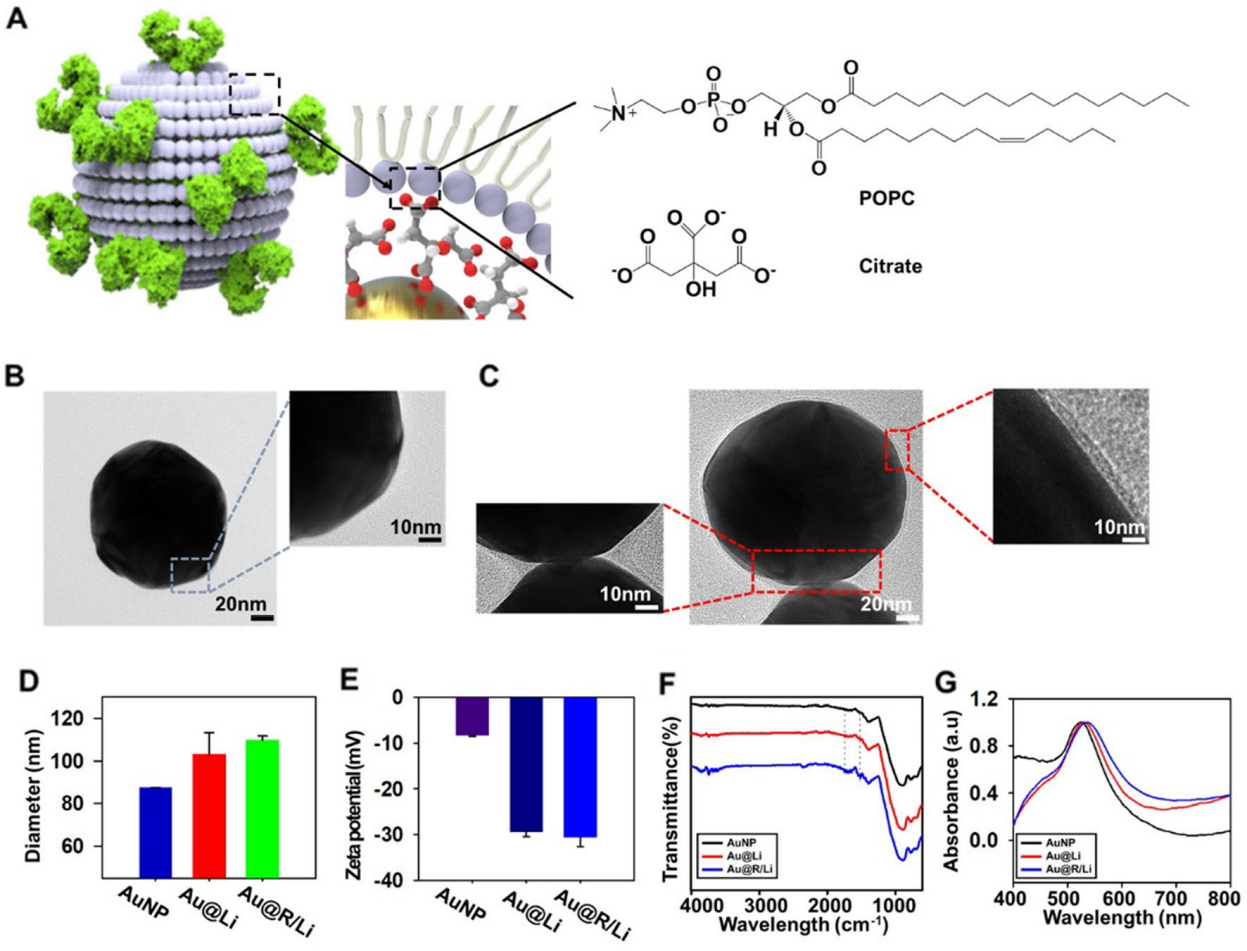
Optimization of Au@R/Li. **A** Schematic image of citrated-coated AuNPs and POPC. **B** Transmission Electron Microscope (TEM) images and magnification of AuNPs and (**C**) Au@Li (scale bars = 20 nm; inset scale bars = 10 nm). **D** DLS size distribution depending on sonication time and the extrusion of AuNPs, Au@Li and Au@R/Li. **E** Surface zeta potentials of AuNPs, Au@Li and Au@R/Li in PBS. **F** Fourier transform infrared spectra of AuNPs, Au@Li and Au@R/Li. **G** Absorbance spectra of AuNPs, Au@Li and Au@R/Li

The interactions between the citrated-coated AuNPs and POPC included electrostatic and van der Waals interactions [31]. Figure 3A shows the chemical structures of POPC and citrate, which coated the AuNPs. The role of the citrate anions in stabilizing the surface of AuNPs is usually related to the formation of a stable conformation during their interaction [32]. In a method of stably dispersing particles by modifying a surface to reduce the agglomeration of AuNPs, a colloidal solution of AuNPs was prepared by adding a solution that underwent a redox reaction to an HAuCl_4_ solution [33]. By using these AuNPs, it is possible to explore the relation between AuNPs and Au@Li with optima ratio (Additional file 1: Fig. S6). To confirm the formation of Au@Li, bare AuNPs and Au@Li were compared by TEM. Figure 3B depicts bare AuNPs (left), measured at 100 nm, and a synthesized liposome, measured at 105 nm. Figure 3C illustrates the process of coating the liposomes on the AuNPs (right). A thin membrane of 5.05 ± 0.78 nm was observed on the surface of the AuNPs, showing that the liposomes covered the AuNPs. In Fig. 3D, size distribution analysis was applied to examine the coating of liposomes onto the AuNPs. Au@R/Li was studied in terms of the changes in AuNP size before and after the fusion process. Following membrane fusion, the diameter of the AuNPs increased from 87 ± 0.17 nm to 103 ± 10.19 nm for Au@Li and to 109 ± 1.94 nm for Au@R/Li. This result indicated that the AuNPs were not aggregated during their interaction with the liposomes. Therefore, the presence or absence of receptors insignificantly affects the size of Au@R/Li. The formation of Au@R/Li was also calculated with surface zeta potential measurements and pH measurements. The zeta potential changed from − 8.37 ± 0.2 mV to − 29.37 ± 1.0 mV with the formation of Au@Li, similar to that of Au@R/Li (− 30.6 ± 2.0 mV) (Fig. 3E). Surface zeta potential measurements were confirmed in PBS buffer (pH 7.4). For pH measurements, a pH meter was used to determine the pH differences between AuNPs, Au@Li, and Au@R/Li. The pH of AuNPs was 6.76 ± 0.05, that of Au@Li was 6.42 ± 0.01, and that of Au@R/Li was 6.53 ± 0.02 in PBS (pH 7.4) (Additional file 1: Fig. S7). The synthesized AuNPs were placed on the substrate, and the constant arrangement was confirmed through SEM imaging (Additional file 1: Fig. S8). Figure 3F is a graph of the Fourier transform infrared (FT-IR) spectra of the bare AuNPs, Au@Li, and the Au@R/Li complex. The different peaks in the spectra were measured at 1530 cm^−1^ for the N–O bond for protein structure, 1735 cm^−1^ for the C = O bond for phospholipids [34, 35]. To validate the formation of Au@R/Li, the intensity of the absorbance peak change was detected with an LSPR sensor. The peaks of the bare AuNPs to Au@Li to Au@R/Li increased from 523 to 530 nm to 534 nm. The absorbance peak of the bare AuNPs shifted from 523 to 530 nm by Au@Li because of the diameter increase by the single layer of the liposome. The movement of the absorbance peak of Au@R/Li is indicated by the 534 nm peak in Fig. 3D and Additional file 1: Fig. S9. Based on these experimental results, we identified the interactions between AuNPs and R/Li and their stable formation through sensing.

### Spike protein of SARS-CoV and SARS-CoV-2 detection of Au@R/Li

**Fig. 4:**
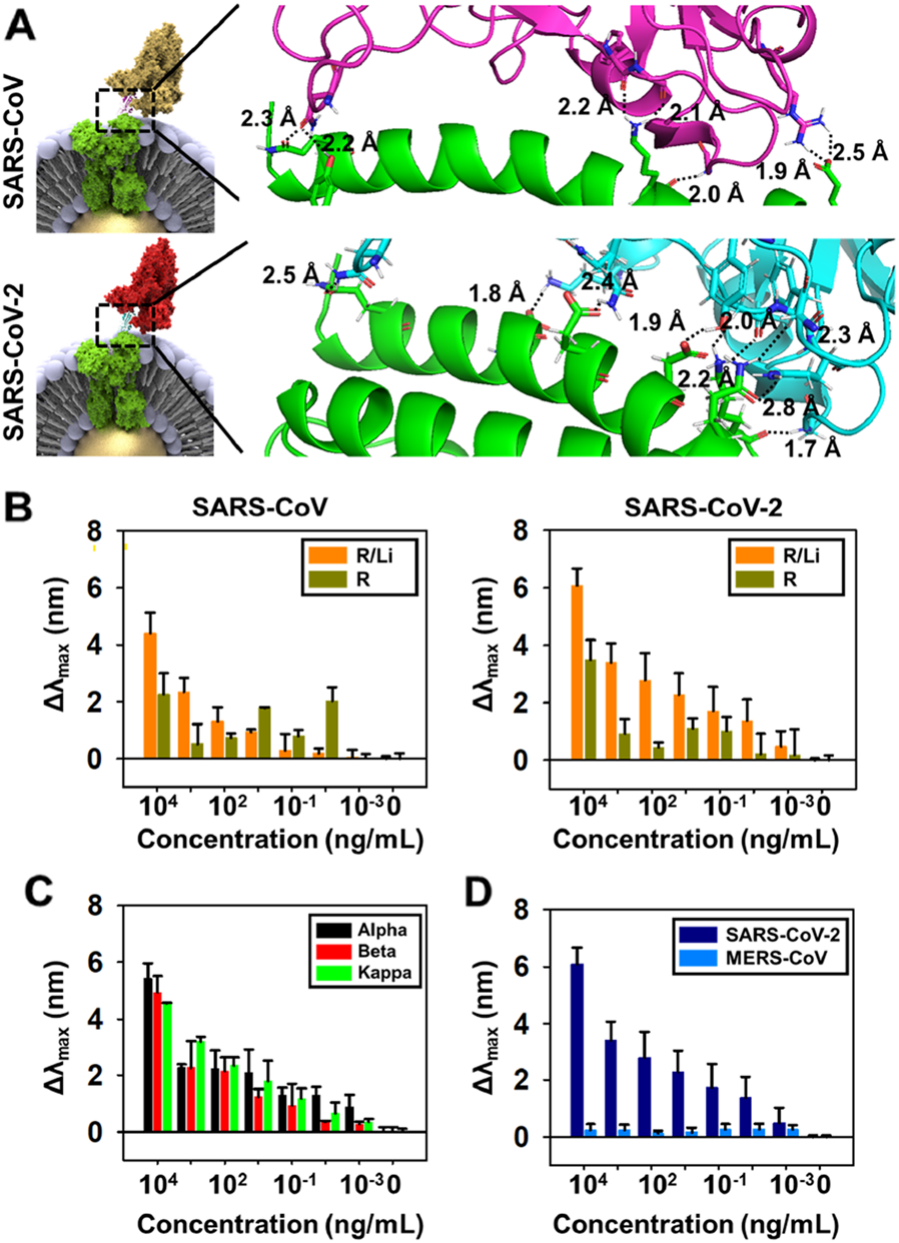
Standard Sample. **A** Schematic image of the interaction between the spike proteins of SARS-CoV (up) and SARS-CoV-2 (down) and R/Li by AutoDock software. **B** Sensitivity graph of the interaction between the spike proteins of SARS-CoV (left) and SARS-CoV-2 (right) on the Au@R and Au@R/Li. **C** Sensitivity of the variant of SARS-CoV-2 (Alpha, Beta, Kappa) with Au@R/Li. **D** Selectivity graph of SARS-CoV-2 and MERS-CoV

Figure 4A shows the simulation results of Au@R/Li, which is a binding spike protein of SARS-CoV and SARS-CoV-2. ACE2 and SARS-CoV spike protein complex (PDB ID: 2AJF) and ACE2 and SARS-CoV-2 spike protein complex (PDB ID: 6M0J) were used for simulation by Autodock software and pymol [36]. The spike protein of SARS-CoV and the ACE2 receptor were activated in GLN 24, TYR 83, LYS 353, and GLU 329, and the spike protein of SARS-CoV-2 and the ACE2 receptor were activated in GLN 24, ASP 30, GLU 35, ASP 38, GLN 42, and LYS 353 for hydrogen bonding. The common residues of ACE2 are GLN 24 and LYS 353, and there is a different distance from SARS-CoV (2.2, 2.3 Å) and SARS-CoV-2 (2.5 Å) to GLN 24 and from SARS-CoV (2.0 Å) and SARS-CoV-2 (1.7 Å) to LYS 353. To determine the interaction between the spike proteins of SARS-CoV and SARS-CoV-2 and Au@R/Li, a wide range of concentrations of spike protein was analyzed for the test (Fig. 4B). In addition, spike proteins were analyzed with ACE2 receptor-functionalized AuNPs without liposomes (Au@R) to indicate the advantage of Au@R/Li. The Au@R was placed on the substrate, and when analyzing the absorbance peak to detect the spike protein, the redshift peak was unstable, regardless of the concentration of spike protein. As shown in Fig. 4B, the results indicated the interaction with viruses (SARS CoV-1 and SARS CoV-2) on Au@R and Au@R/Li, respectively. First, Au@R and Au@R/Li non-treated with viruses has not shown the peak, and then the interaction between the Au@R and Au@R/Li treated with viruses was analyzed through the observation of shifted peak. The spike protein of SARS CoV-1 and SARS CoV-2 on Au@R was randomly detected on low concentration (0.01 ~ 10 ng/ml) due to the non-specific binding of ACE 2 receptor and binding site of spike protein. Whereas, the detection wavelength of the SARS-CoV-1 and SARS-CoV-2 on Au@R/Li was significantly increased according to increase of virus concentration. In addition, SARS CoV-1 with 10,000, 1000, and 100 ng/ml concentration were increased the wavelength in Au@R/Li to the 1.95, 4.51 and 1.78-fold compared that SARS CoV-1 detected on Au@R surface, respectively. Similarly, the SARS CoV-2 detection on Au@R/Li shown increasing to 1.68 ~ 6.16-fold in 0.01 ~ 10,000 ng/ml concentration compared to Au@R. Also, LOD of viruses on Au@R/Li was 0.01 ng/ml concentration. These results shown that the Au@R/Li increased the detection sensitivity of spike proteins with various ranges. Furthermore, the detection of spike protein variant was conducted using an Au@R/Li. The requirement of spike protein variant detection has been continuing by emerging of new variants. The sensitivity and selectivity performances were conducted with alpha, beta, and kappa variants, as shown in Fig. 4C. Au@R/Li detected the alpha, beta, and kappa variants of the spike protein, which interact with the ACE2 receptor to penetrate the host cell. In addition to the normal spike protein, variants, the sensitivity of the binding receptor to the binding domain of the variants of the spike protein was analyzed. As with the normal spike protein of SARS-CoV-2, variant spike proteins of SARS-CoV-2 were also detected by ACE2 proportionately with the concentration of spike protein. To validate the selectivity of the ACE2 receptor, the MERS-CoV spike protein was used (Fig. 4D). While SARS-CoV-2 and MERS-CoV are members of the betacoronavirus family, the spike protein of MERS-CoV does not target the ACE2 receptor [37]. Due to the difference in spike protein, MERS-CoV did not bind with Au@R/Li.

### COVID-19 detection in clinical samples of Au@R/Li

**Fig. 5:**
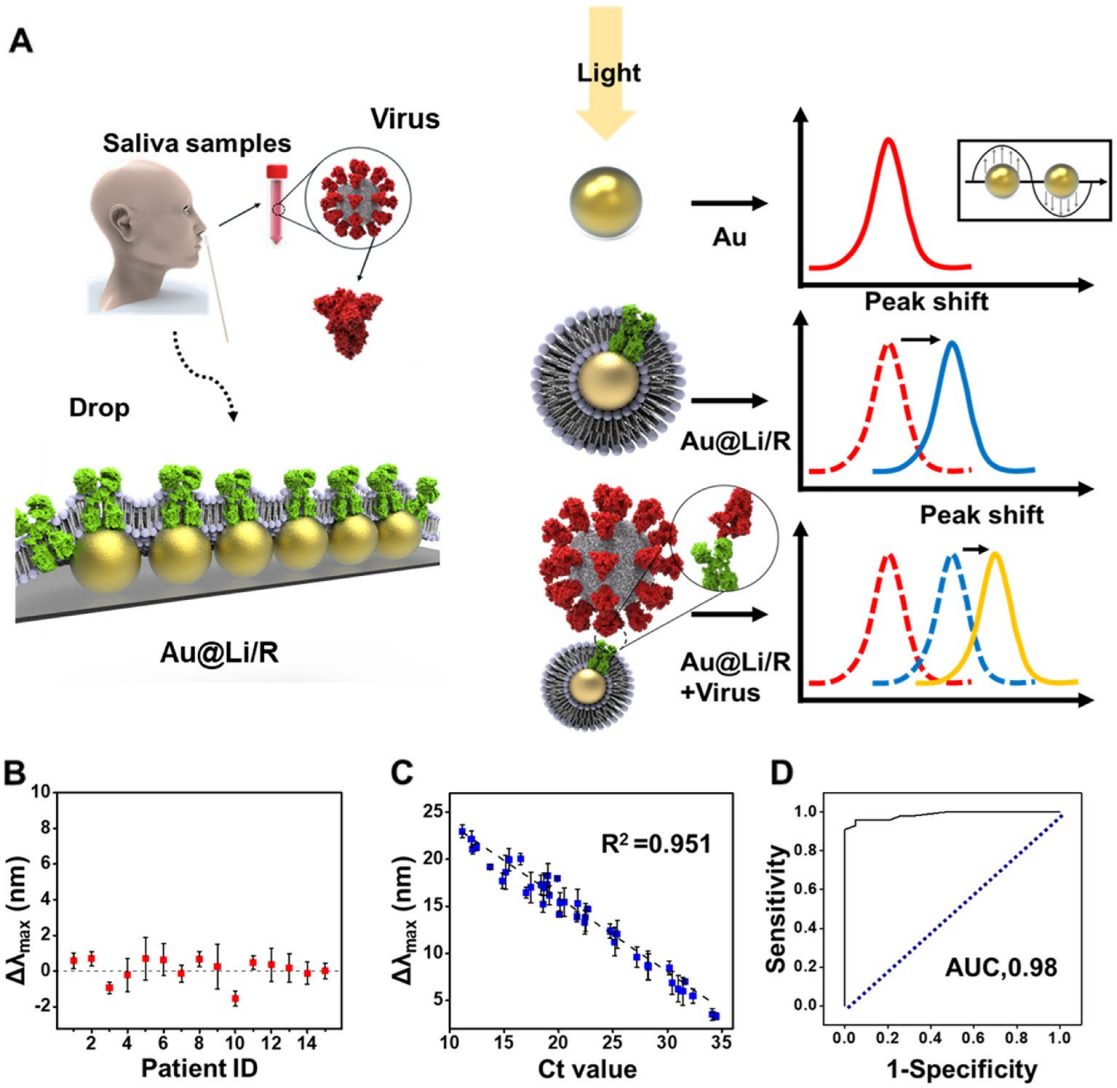
Clinical sample of the saliva of COVID-19 patients. **A** Schematic image of the mechanism for clinical sample detection with Au@R/Li by the LSPR sensor. Sensitivity graph of the (**B**) negative control group and (**C**) COVID-19 patient group. **D** ROC curve of the validation test results for a comparison of the negative control and COVID-19-positive patient samples

Figure 5A schematically depicts the clinical sample detection performance of the LSPR sensor. For other sensors, a pretreatment process, such as RNA extraction, is needed, but our LSPR did not require pretreatment because the spike protein was detected by the ACE2 receptor. After the collection of clinical samples from the UTM, the saliva samples contained in the UTM were dropped onto the LSPR sensor for detection. However, UTM includes various reagents. A control experiment in PBS and UTM for zeta potential analysis for sensor formation during detection is schematically depicted in Additional file 1: Fig. S10, and the corresponding pH measurements are shown in Additional file 1: Fig. S4. When R/Li was placed on the AuNPs and SARS-CoV-2 was detected using it, a peak shift occurred gradually. The signals of the samples were stable given that the numerical values did not fluctuate significantly. To facilitate a comparison to the samples of patients with or without SARS-CoV-2, a negative control experiment was conducted by collecting samples from patients who had COVID-19 results that were negative by PCR. As confirmed in the graph, there was no absorbance peak shift (Fig. 5B). As shown in Fig. 5C, as the Ct value increased, the redshift value inversely decreased. A linear fitting curve of the Ct value was plotted. In the range of 10 to 35 Ct values from patients, the LSPR sensor detected a broad concentration range of Ct values. From these data, it was concluded that the LSPR sensor detected SARS-CoV-2 because there was a shift change even when the Ct value reached 25 ~ 31. By comparing these data with the commercially used LFA kit results, it was observed that the Ct value increased from 20 to more, and the LFA kit did not detect positivity, but the LSPR sensor detected positivity (Additional file 1: Fig. S11). The Au@R/Li shows a 98% accuracy of sensitivity according to the receiver operating characteristic (ROC) curve, which was calculated from 40 COVID-19-positive samples and 15 negative control samples. Compared with conventional SARS-CoV-2 diagnosis sensors, a potential advantage of LSPR sensors is the detection of a wide range of Ct values for clinical samples, as shown in Additional file 1: Table S1. As a result, the LSPR sensor detected SARS-CoV-2 spike protein in clinical samples.

## Conclusion

We developed a virus detection platform with LSPR method that can detect easily, accurately, and rapidly without preprocessing clinical samples using liposome technology for potentially innovative materials. The recombinant protein reconstituted with liposomes was used for detecting SARS-CoV and SARS-CoV-2. We successfully manipulated host receptor, ACE2, with liposomes, which were embedded between liposomes for high sensitivity and stability because individual proteins are denaturized, unfolded, and lost functional activity due to their unstable form. Also compared to using antibodies in liposomes, recombinant protein in liposome has higher affinity. We calculated binding affinity and simulated expectation between antibody and recombinant protein by simulation.

The recombinant protein embedded liposomes were assembled on AuNPs, and LSPR method was used for detection. With the sensor platform S1 protein of both viruses was detected with detection limit of 10 pg/ml. Our platform detected clinical data from 40 people with Ct values from 10 to 35, and a range of peak shifting from 5 to 25 nm was observed successfully. In particular, the reconstitution of ACE2 into liposomes allowed the analysis of clinical samples with high Ct values, low concentrations of spike protein, and early stages of viral infection. We confirmed that the detection limitation of Au@R/Li significantly increased compared to that of Au@R. In the selectivity test, SARS-CoV and SARS-CoV-2 showed a signal when binding with Au@R/Li, but MERS-CoV did not show a signal. The proposed sensor platform can be used as promising detection method with high sensitivity and selectivity for the early and simple diagnosis of new emerging viruses.

### Supplementary Information

**Additional file 1: Figure S1.** Formation of phospholipids on AuNPs. **Figure S2.** Coomassie Blue image of Lipid:Receptor mix buffer. **Figure S3.** Comparison of Liposome before sonication/extrusion and after. **Figure S4.** Simulation of ACE2 with POPC and POPG by Ligplot. **Figure S5.** Simulation of antibodies with POPC and POPG membrane by CHARMM-GUI, POPC and POPG binding affinity by Autodock. **Figure S6.** Absorbance spectra of Au@R/Li with ratio (A) 0.05 (v/v%) (B) 0.01 (v/v%) (c) 0.005 (v/v%) (d) 0.001 (v/v%) (up), inlet (down). **Figure S7.** pH measurement of AuNPs, Au@Li and Au@R/Li in PBS and UTM. **Figure S8.** SEM analysis of AuNPs. **Figure S9.** Absorbance spectra of AuNPs, Au@Li and Au@R/Li. **Figure S10.** Surface zeta potentials of AuNPs, Au@Li and Au@R/Li in UTM; **Figure S11.** Comparison with LFA kit and LSPR sensor to detect SARS-CoV-2; and **Table S1.** Comparison of sensors to detect SARS-CoV-2 virus S protein by using ACE2 (PDF).

## Data Availability

The datasets used and/or analysed during the current study are available from the corresponding author on reasonable request.

## References

[1] World Health Organization, Weekly epidemiological update on COVID-19 - 23 November 2022. https://www.who.int/publications/m/item/weekly-epidemiological-update-on-covid-19---23-november-2022. Accessed 11 April 2023.

[2] Naqvi AAT, Fatima K, Mohammad T, Fatima U, Singh IK, Singh A, Atif SM, Hariprasad G, Hasan GM, Hassan MI (2020). Biochim. Biophys. Acta. Mol. Basis Dis..

[3] World Health Organization, WHO to identify pathogens that could cause future outbreaks and pandemics. https://www.who.int/news/item/21-11-2022-who-to-identify-pathogens-that-could-cause-future-outbreaks-and-pandemics. Accessed 11 May 2023.

[4] Gremmels H, Winkel BMF, Schuurman R, Rosingh A, Rigter NAM, Rodriguez O, Ubijaan J, Wensing AMJ, Bonten MJM, Hofstra LM (2021). EClinicalMedicine.

[5] Grant BD, Anderson CE, Williford JR, Alonzo LF, Glukhova VA, Boyle DS, Weigl BH, Nichols KP (2020). Anal. Chem..

[6] Tastanova A, Stoffel CI, Dzung A, Cheng PF, Bellini E, Johansen P, Duda A, Nobbe S, Lienhard R, Bosshard PP, Levesque MP (2021). J. Mol. Diagnostics.

[7] Seo G, Lee G, Kim MJ, Baek S-H, Choi M, Ku KB, Lee C-S, Jun S, Park D, Kim HG, Kim S-J, Lee J-O, Kim BT, Park EC, Kim SI (2020). ACS Nano.

[8] Miller EH, Obernosterer G, Raaben M, Herbert AS, Deffieu MS, Krishnan A, Ndungo E, Sandesara RG, Carette JE, Kuehne AI, Ruthel G, Pfeffer SR, Dye JM, Whelan SP, Brummelkamp TR, Chandran K (2012). EMBO J..

[9] Deng F, Ye G, Liu Q, Navid MT, Zhong X, Li Y, Wan C, Xiao S, He Q, Fu ZF, Peng G (2016). Viruses.

[10] Van Der Hoek L, Pyrc K, Jebbink MF, Vermeulen-Oost W, Berkhout RJM, Wolthers KC, Wertheim-Van Dillen PME, Kaandorp J, Spaargaren J, Berkhout B (2004). Nat. Med..

[11] Yuan M, Wu NC, Zhu X, Lee CCD, So RTY, Lv H, Mok CKP, Wilson IA (2020). Science.

[12] Hoffmann M, Kleine-Weber H, Schroeder S, Krüger N, Herrler T, Erichsen S, Schiergens TS, Herrler G, Wu NH, Nitsche A, Müller MA, Drosten C, Pöhlmann S (2020). Cell.

[13] Jia H, Neptune E, Cui H (2021). Am. J. Respir. Cell Mol. Biol..

[14] Lavington S, Watts A (2020). Biophys. Rev..

[15] Akbarzadeh A, Rezaei-Sadabady R, Davaran S, Joo SW, Zarghami N, Hanifehpour Y, Samiei M, Kouhi M, Nejati-Koshki K (2013). Nanoscale Res. Lett..

[16] Bozzuto G, Molinari A (2015). Int. J. Nanomedicine.

[17] Daraee H, Etemadi A, Kouhi M, Alimirzalu S, Akbarzadeh A (2016). Artif. Cells. Nanomed. Biotechnol..

[18] Singh P, Pandit S, Mokkapati VRSS, Garg A, Ravikumar V, Mijakovic I (2018). Int. J. Mol. Sci..

[19] Zhang X, Guo Q, Cui D (2009). Sensors.

[20] Friedman A, Claypool S, Liu R (2013). Curr. Pharm. Des..

[21] Lim SG, Jo S, Lee JH, Kwon OS (2022). Appl. Sci. Converg. Technol..

[22] Homola J (2008). Chem. Rev..

[23] Huang Y, Yang C, Feng Xu X, Xu W, Wen Liu S (2020). Acta Pharmacol. Sin..

[24] Ciancaglini P, Simão AMS, Bolean M, Millán JL, Rigos CF, Yoneda JS, Colhone MC, Stabeli RG (2012). Biophys. Rev..

[25] Qiu W, Fu Z, Xu GG, Grassucci RA, Zhang Y, Frank J, Hendrickson WA, Guo Y (2018). Proc. Natl. Acad. Sci..

[26] Rosano GL, Ceccarelli EA (2014). Front. Microbiol..

[27] Cuatrecasas P (1970). J. Biol. Chem..

[28] Jo S, Kim T, Iyer VG, Im W (2008). J. Comput. Chem..

[29] Wu EL, Cheng X, Jo S, Rui H, Song KC, Dávila-Contreras EM, Qi Y, Lee J, Monje-Galvan V, Venable RM, Klauda JB, Im W (2014). J. Comput. Chem..

[30] Trott O, Olson AJ (2010). J. Comput. Chem..

[31] Contini C, Hindley JW, Macdonald TJ, Barritt JD, Ces O, Quirke N (2020). Commun. Chem..

[32] Wang B, Zhang L, Bae SC (2008). S. Granick. Proc. Natl. Acad. Sci..

[33] Yeh YC, Creran B, Rotello VM (2012). Nanoscale.

[34] Hwang J, An E-K, Kim S-J, Zhang W, Jin J-O (2022). ACS Nano.

[35] Ami D, Lavatelli F, Rognoni P, Palladini G, Raimondi S, Giorgetti S, Monti L, Doglia SM, Natalello A, Merlini G (2016). Sci. Rep..

[36] Lan J, Ge J, Yu J, Shan S, Zhou H, Fan S, Zhang Q, Shi X, Wang Q, Zhang L, Wang X (2020). Nature.

[37] Wang N, Shi X, Jiang L, Zhang S, Wang D, Tong P, Guo D, Fu L, Cui Y, Liu X, Arledge KC, Chen YH, Zhang L, Wang X (2013). Cell Res..

