# Supplementary material for: Recombinant protein embedded liposome on gold nanoparticle based on LSPR method to detect Corona virus
Source: Nano Converg. 2023 Oct 30;10:51. doi: 10.1186/s40580-023-00399-x (PMC10615991; doi:10.1186/s40580-023-00399-x)
Supplement: Supplementary file 1 — Additional file 1: Figure S1. Formation of phospholipids on AuNPs. Figure S2. Coomassie Blue image of Lipid:Receptor mix buffer. Figure S3. Comparison of Liposome before sonication/extrusion and after. Figure S4. Simulation of ACE2 with POPC and POPG by Ligplot. Figure S5. Simulation of antibodies with POPC and POPG membrane by CHARMM-GUI, POPC and POPG binding affinity by Autodock. Figure S6. Absorbance spectra of Au@R/Li with ratio (A) 0.05 (v/v%) (B) 0.01 (v/v%) (c) 0.005 (v/v%) (d) 0.001 (v/v%) (up), inlet (down). Figure S7. pH measurement of AuNPs, Au@Li and Au@R/Li in PBS and UTM. Figure S8. SEM analysis of AuNPs. Figure S9. Absorbance spectra of AuNPs, Au@Li and Au@R/Li. Figure S10. Surface zeta potentials of AuNPs, Au@Li and Au@R/Li in UTM; Figure S11. Comparison with LFA kit and LSPR sensor to detect SARS-CoV-2; and Table S1. Comparison of sensors to detect SARS-CoV-2 virus S protein by using ACE2 (PDF). [file 40580_2023_399_MOESM1_ESM.docx]

**Recombinant protein embedded liposome on gold nanoparticle based on LSPR method to detect Corona virus**

Lina Kim^a,b, ‡^, Seongjae Jo^a, ‡^_,_ Gyeong-Ji Kim^d, ‡^, Kyung Ho Kim^a^, Sung Eun Seo^a^, Eunsu Ryu^a^, Chan Jae Shin^d^ ,Yu Kyung Kim^c^, Jeong-Woo Choi^b,*^, Oh Seok Kwon^a,d,*^

^a^ Infectious Disease Research Center, Korea Research Institute of Bioscience and Biotechnology (KRIBB), Daejeon 34141, Republic of Korea

^b^ Department of Chemical & Biomolecular Engineering, Sogang University, 35 Baekbeom-Ro, Mapo-Gu, Seoul 04107, Republic of Korea

^c^ Department of Clinical Pathology, School of Medicine, Kyungpook National University, 80, Daehak-ro, Buk-gu, Daegu, Korea

^d^ SKKU Advanced Institute of Nanotechnology (SAINT), Department of Nano Engineering, Sungkyunkwan University (SKKU), Suwon, 16419 Republic of Korea

* Corresponding author.

Tel: +82-2-705-8480; Fax: +82-2-3273-0331; E-Mail addresses: [jwchoi@sogang.ac.kr](mailto:jwchoi@sogang.ac.kr) (Jeong-Woo Choi),

Tel: +82-42-860-8284; Fax: +82-42-879-8594; E-Mail addresses: [oskwon79@skku.edu](mailto:oskwon79@skku.edu), [oskwon7799@gmail.com](mailto:oskwon7799@gmail.com) (Oh Seok Kwon)

^‡^These authors contributed equally

**EXPERIMENTAL SECTION**

**Supporting results**

**Figure S1**


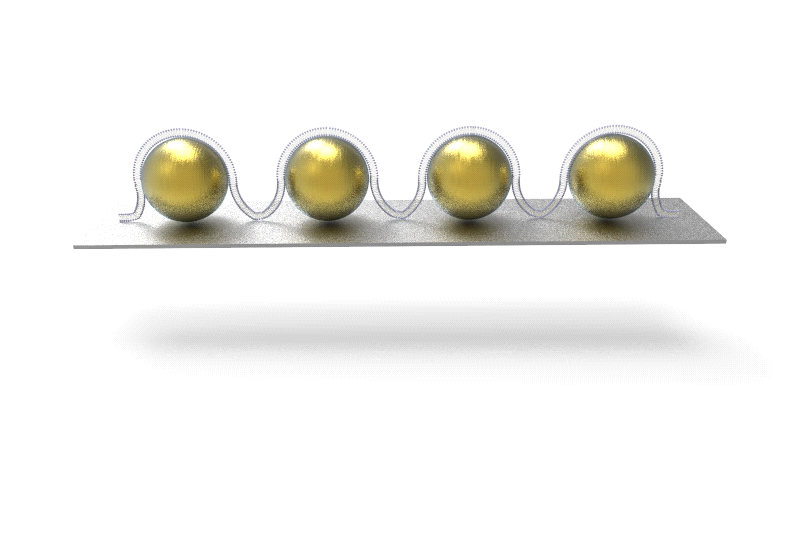


**Figure S1**. Formation of phospholipids on AuNPs.

**Figure S2**


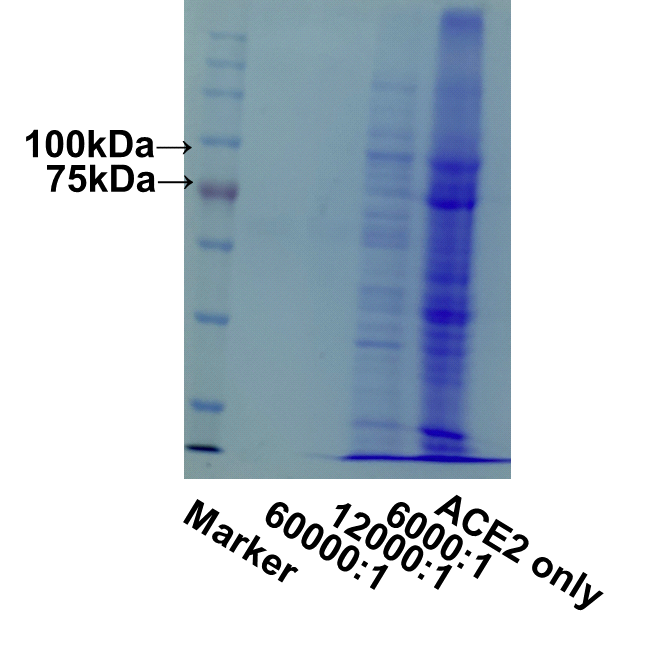


**Figure S2**. Coomassie Blue image of Lipid:Receptor mix buffer.

**Figure S3**


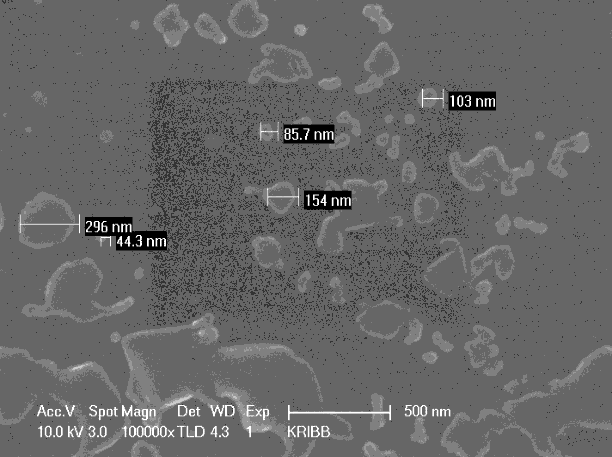

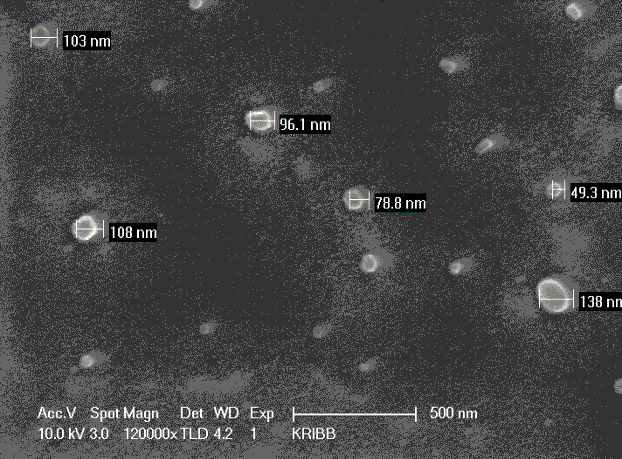


**Figure S3**. Comparison of Liposome before sonication/extrusion (left) after (right).

**Figure S4**


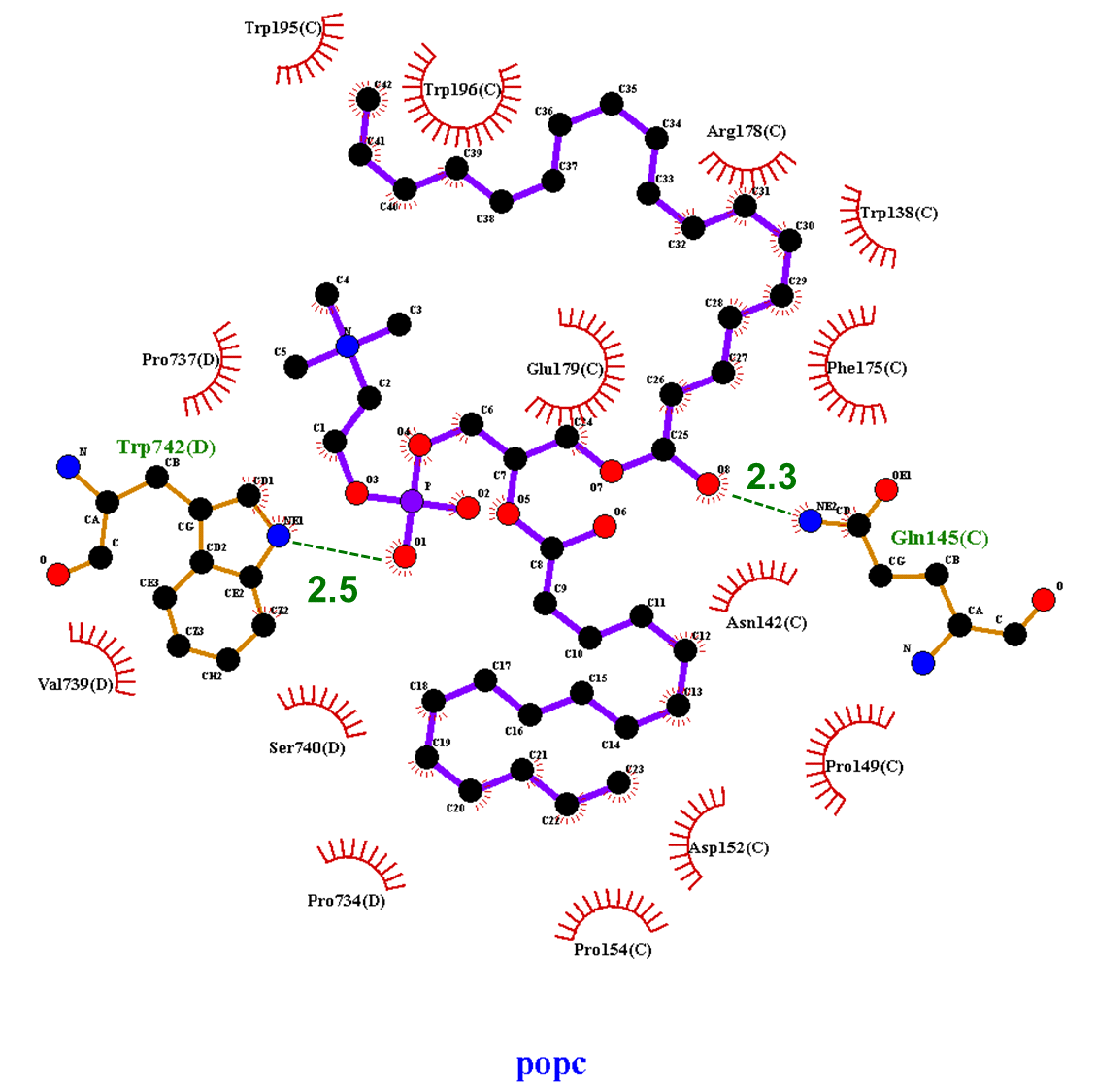

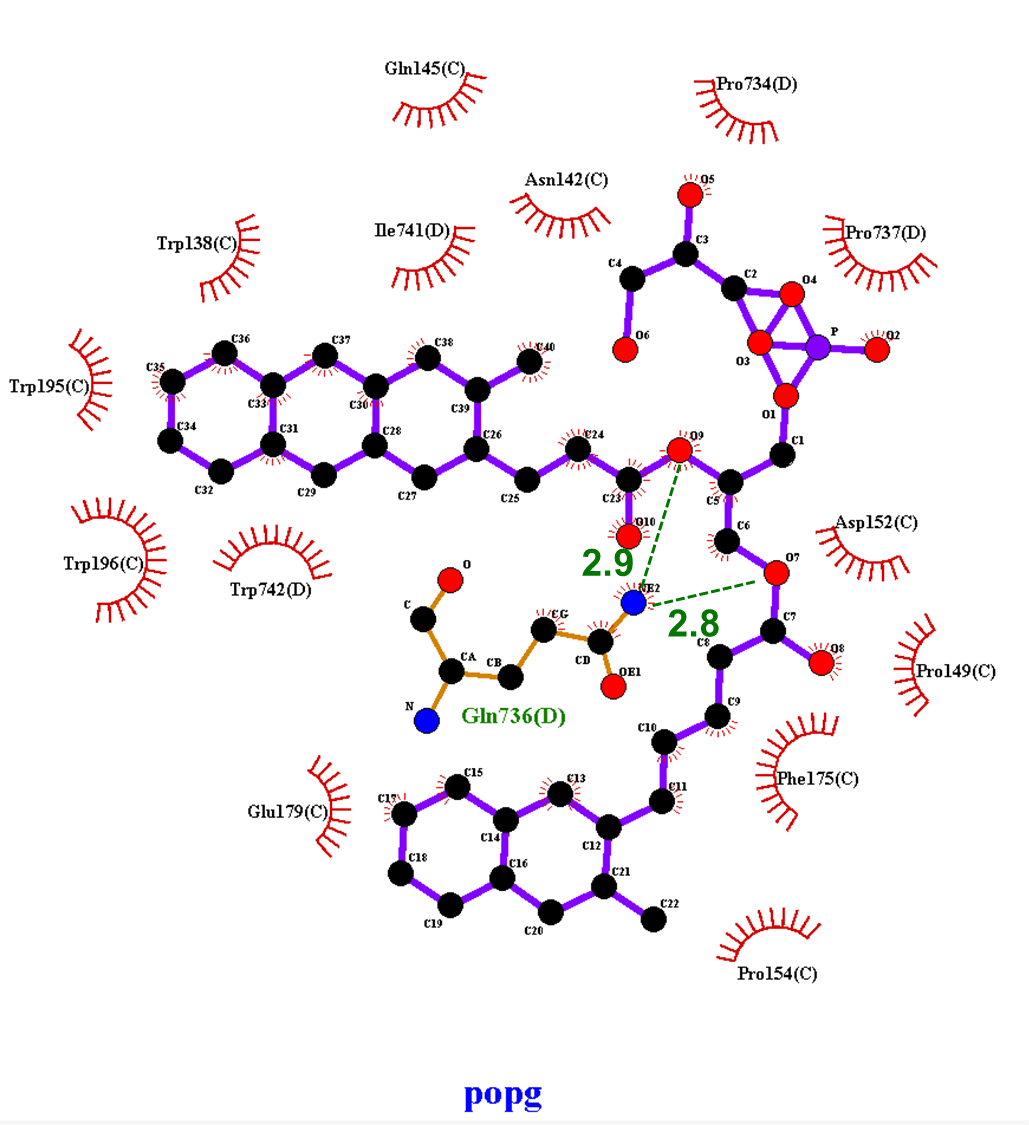


**Figure S4.** Simulation of ACE2 with 1-palmitoyl-2-oleoyl-sn-glycero-3-phosphocholine (POPC) (left)and 1-palmitoyl-2-oleoyl-sn-glycero-3-(phospho-rac-(1-glycerol) (POPG)(right) by Ligplot.(Green line = Hydrogen bond, Red line = hydrophobic interaction)

**Figure S5**


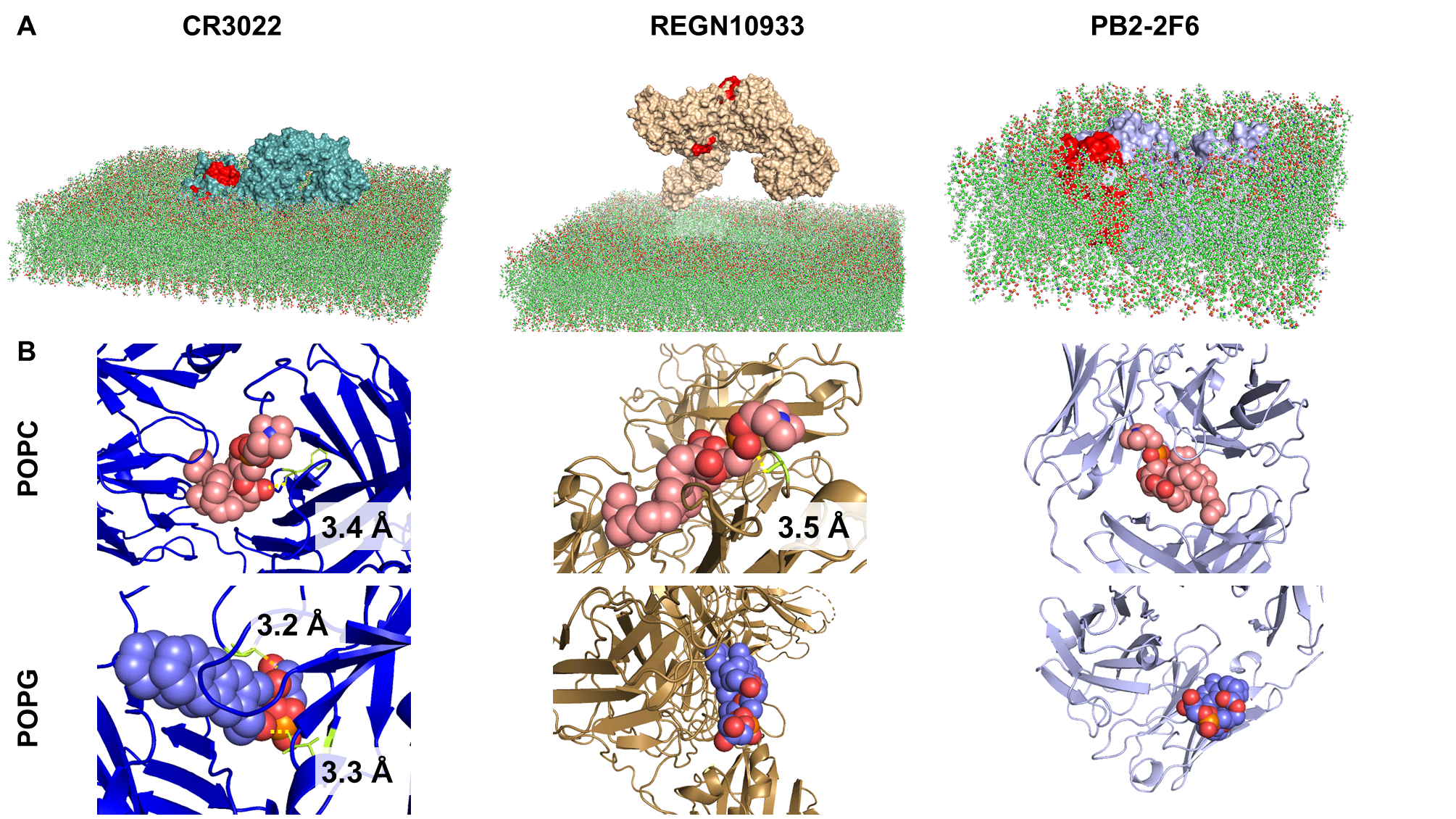


**Figure S5.** Simulation of antibodies with (A) POPC and POPG membrane by Charmm-Gui , (B) POPC and POPG binding affinity by Autodock.

**Figure S6**


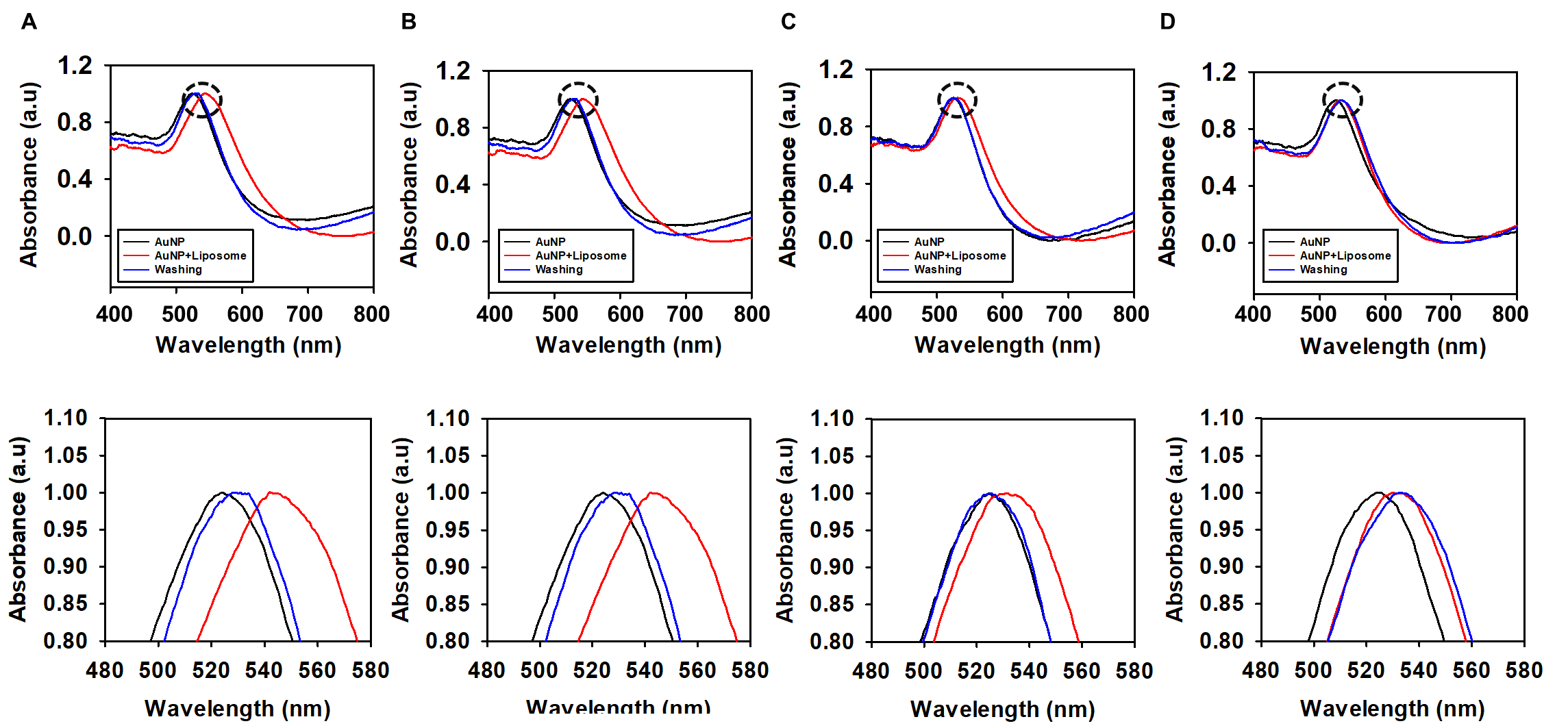


**Figure S6.** Absorbance spectra of Au@R/Li with ratio (A) 0.05 (v/v%) (B) 0.01 (v/v%) (c) 0.005 (v/v%) (d) 0.001 (v/v%) (up), inlet (down)

**Figure S7**


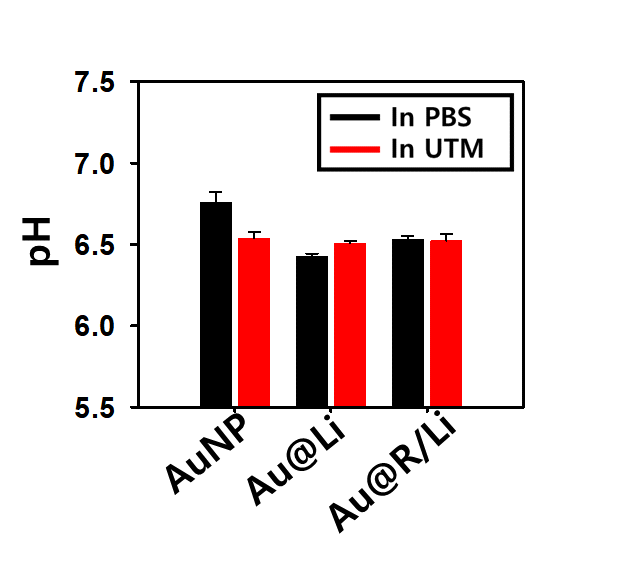


**Figure S7.** pH measurement of AuNPs, Au@Li and Au@R/Li in PBS and UTM.

**Figure S8**


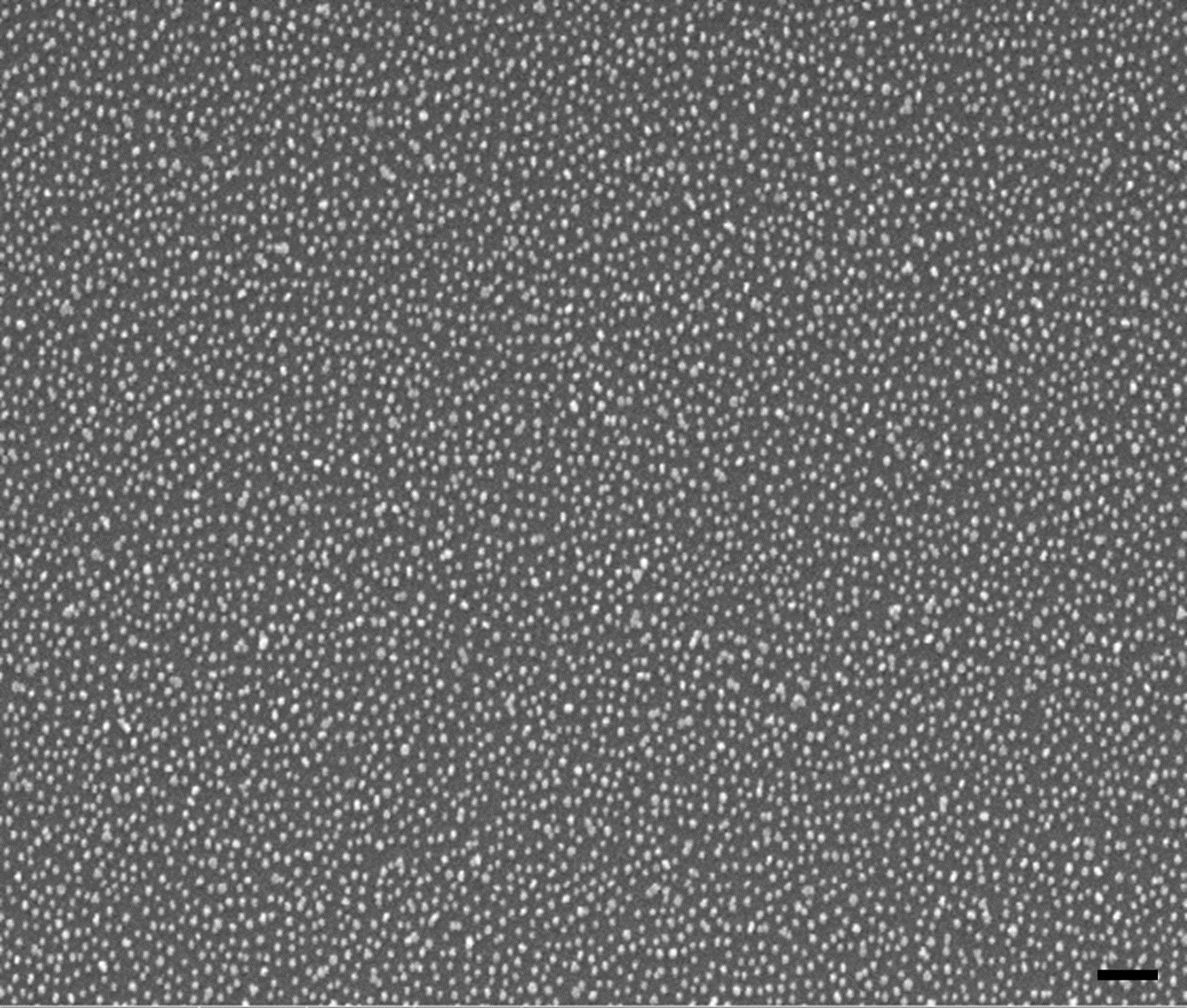


**Figure S8.** SEM analysis of AuNPs. (Scale bar = 500 nm)

**Figure S9**


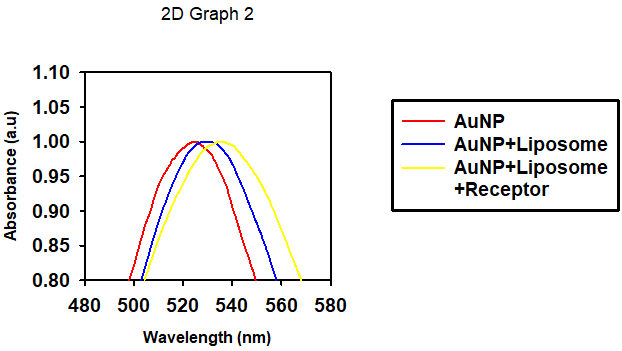


**Figure S9.** Absorbance spectra of AuNPs, Au@Li and Au@R/Li.

**Figure S10**


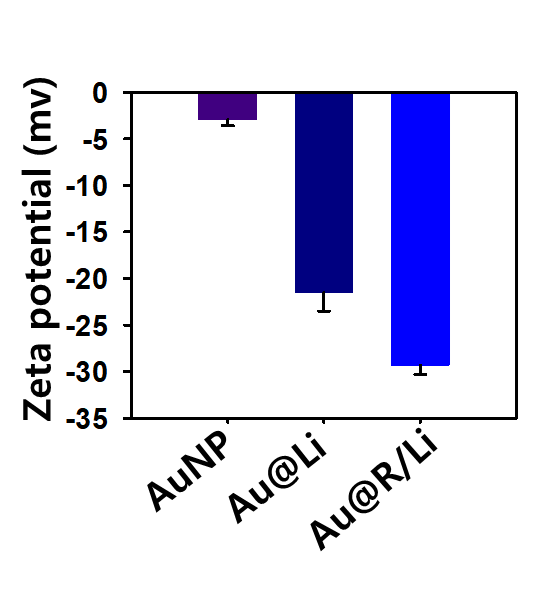


**Figure S10.** Surface zeta potentials of AuNPs, Au@Li and Au@R/Li in UTM.

**Figure S11**


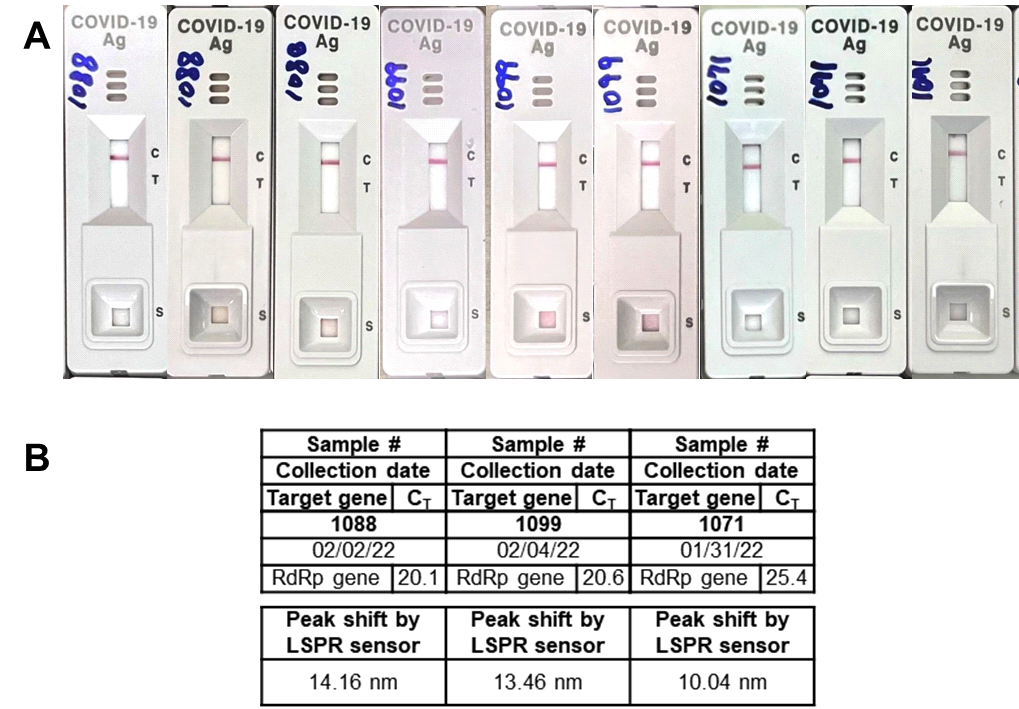


**Figure S11.** Comparison with LFA kit and LSPR sensor to detect SARS-CoV-2. (A) Detection of Ct value > 20 SARS-CoV-2 viruses by LFA kit. (B) Detection of Ct value >20 SARS-CoV-2 viruses by LSPR sensor.

**Table S1.** Comparison of sensors to detect SARS-CoV-2 virus S protein by using ACE2.

| Name | Limit of detection | Sample  preparation step | Additional information | Reference |
| --- | --- | --- | --- | --- |
| Silicon nanowire biosensor | 100 ng/ml | X | Detection by current-voltage characteristics | Gao, Bingtao, et al.,2022 |
| Carbon Nanotube  biosensor | 35 mg/L | X | Targeted to RBD domain of Spike protein | Pinals, Rebecca L., et al.,2021 |
| ACE2-based LFIA | 5 ng/ml | X | - | Lee, Jong-Hwan, et al.,2021 |
| Colorimetric peptide pair sensor | 0.26 ng/mL | X | Detection in water environment | Zhu, Qian, and Xiaohong Zhou.,2021 |

**Reference**

- B. Gao, R.A.R. Chavez, W. I. Malkawi, D. W. Keefe, R. Smith, H. Haim, A. K. Salem, F. Toor, Sens. Biosensing. Res. **36,** 100487 (2022)
- R. L Pinals, F. Ledesma, D. Yang, N. Navarro, S. Jeong, J.E. Pak, L. Kuo, Y.-W. Cheng, H.-Y. Sun, M.P. Landry, Nano Lett. **21,** 2272-2280 (2021)
- J.-H. Lee, M. Choi, Y. Jung, S.K. Lee, C.-S. Lee, J. Kim, J. Kim, N.H. Kim, B.-T. Kim, H.G. Kim, Biosens. Bioelectron. **171,** 112715 (2021)
- Q. Zhu, X. Zhou, J. Hazard. Mater. **425,** 127923 (2022)
- R.A. Laskowski, M. B. Swindells, J. Chem. Inf. Model. **51,** 2778-2786 (2011)
- S. Jo, T. Kim, V.G. Iyer, W. Im, J. Comput. Chem. **29,** 1859–1865 (2008)
- C. Kim, D.-K. Ryu, J. Lee, Y.-I. Kim, J.-M. Seo, Y.-G. Kim, J.-H. Jeong, M. Kim, J.-I. Kim, P. Kim, J.S. Bae, E.Y. Shim, M.S. Lee, M. S. Kim, H. Noh, G.-S. Park, J.S. Park, D. Son, Y. An, J.N. Lee, K.-S. Kwon, J.-Y. Lee, H. Lee, J.-S. Yang, K.-C. Kim, S.S. Kim, H.-M. Woo, J.-W. Kim, M.-S. Park, K.-M. Yu, S.-M. Kim, E.-H. Kim, S.-J. Park, S.T. Jeong, C.H. Yu, Y. Song, S.H. Gu, H. Oh, B.-S. Koo, J.J. Hong, C.M. Ryu, W.B. Park, M.-D. Oh, Y.K. Choi, S.-Y. Lee, Nat. Commun. **12,** 288 (2021)
- B. Ju, Q. Zhang, J. Ge, R. Wang, J. Sun, X. Ge, J. Yu, S. Shan, B. Zhou, S. Song, X. Tang, J. Yu, J. Lan, J. Yuan, H. Wang, J. Zhao, S. Zhang, Y. Wang, X. Shi, L. Liu, J. Zhao, X. Wang, Z. Zhang, L. Zhang, Nature. **584** 115-119 (2020)
- M. Yuan, N. C. Wu, X. Zhu, C.C.D. Lee, R.T.Y. So, H. Lv, C.K.P. Mok, I.A.A. Wilson, Science **368,** 630-633 (2020)
